# Research Progress on the Animal Models of Drug-Induced Liver Injury: Current Status and Further Perspectives

**DOI:** 10.1155/2019/1283824

**Published:** 2019-04-15

**Authors:** Yingying Pan, Mingzhu Cao, Danming You, Genggeng Qin, Zhi Liu

**Affiliations:** ^1^Department of Ultrasound, Nanfang Hospital, Southern Medical University, Guangzhou, China; ^2^Department of Obstetrics and Gynecology, Sun Yat-sen Memorial Hospital, Sun Yat-sen University, Guangzhou, China; ^3^Department of Radiology, Nanfang Hospital, Southern Medical University, Guangzhou, China

## Abstract

Drug-induced liver injury (DILI) is a major concern in clinical studies as well as in postmarketing surveillance. It is necessary to establish an animal model of DILI for thorough investigation of mechanisms of DILI and searching for protective medications. This article reviews the current status and future perspective on establishment of DILI models based on different hepatotoxic drugs, as well as the underlying mechanisms of liver function damage induced by specific medicine. Therefore, information from this article can help researchers make a suitable selection of animal models for further study.

## 1. Introduction

Drug-induced liver injury (DILI) refers to acute or chronic liver injury induced by usage of certain medications and/or by their metabolites. DILI has become the most common cause of acute liver injury nowadays and also accounts for around one in ten cases of adverse drug reactions [1]. The incidence of DILI has been reported to be between 14 and 20 per 100 000 patients [2]. Serious liver injury may lead to liver failure or even be life threatening [3]. Therefore, DILI should be taken seriously by clinicians and the public.

There are several classification systems of DILI. Generally, DILI can be divided into two subtypes. The first subtype of liver injury involves direct injury on structure and function of hepatocytes by the medicine itself or its metabolites. The other subtype is more complicated, in which hepatotoxicity is mainly due to oversensitization of liver cells to damages induced by cytokines [4]. Another commonly recognized classification subcategorized DILI into dose-dependent and dose-independent DILI [5]. The latter one is also named idiosyncratic DILI (iDILI) [6].

The hepatotoxicity by DILI is a complex procedure. The major cellular changes involve hepatocytes apoptosis, as well as death of cholangiocytes and endothelial cells [7]. However, due to scanty knowledge of the mechanisms of DILI, standard criteria for diagnosis and effective management are not established yet. Therefore, further understanding of the underlying mechanisms of DILI is crucial.

Animal model for investigating DILI is one critical step of preclinical researches. This is particularly true since the modulation of immune system on development of DILI cannot be achieved in the* in vitro* researcher based on cells and tissues. Both rodents and nonrodents models are available in DILI research field. No clear evidences showed that nonrodent models are superior to rodent model considering the physiological resemblance to human body; thus, this manuscript focused on rodent models due to its easy access in laboratory.

Among all these emerging models that have been developed, which one is to be chosen for investigation of specific drug and how to establish a DILI model? Here, in this review, we will summarize and compare the characteristics and applications of different animal models of DILI based on hepatotoxic drugs, as well as the underlying mechanisms of liver function damage, to help researchers make a suitable selection of animal models for further study.

## 2. Animal Model of DILI Established by Nonsteroidal Anti-Inflammatory Drugs (NSAID)

NSAID are one of the most commonly used over-the-counter drugs and are prescribed for relief of pain, fever, and inflammation. Acetaminophen (APAP), also known as paracetamol, is one of the most widely used NSAID. It is safe and effective when used at therapeutic dose. However, due to excessive use of APAP, severe liver damage can be induced in both experimental animals and humans [8], which accounts for approximately half of the cases of DILI in the United States [9]. Therefore, APAP is widely used to establish DILI models thanks to its easy access and simple operation. This model is used to study the mechanism of DILI and test the effectiveness of drugs against APAP-induced hepatotoxicity.

A single intraperitoneal injection of 300 to 500 mg/kg APAP into mice can produce the DILI model [10, 11]. It is recommended that mice of the same age should be used in studies related to APAP-induced liver injury and that strict standardized operation protocols (SOPs) in animal experimentation should be established [12].

Mechanisms for APAP hepatotoxicity have been extensively investigated. Mitochondrial dysfunction and oxidative stress actively contribute to APAP-induced hepatotoxicity. Toxic doses of APAP produce excessive* N*-acetyl-*p*-benzoquinoneimine (NAPQI) and consume glutathione (GSH), which can damage the function of mitochondria and thus increase the production of mitochondrial reactive oxygen species (mROS), resulting in oxidant stress. Consequently, mitogen-activated protein kinase (MAPK) and downstream c-Jun N-terminal kinase (JNK) activation further impair mitochondrial function, amplify the oxidative stress that inactivates caspases, and promote necrosis [13–15]. While the exact mechanisms remain unclear, most studies consider it as a compensatory response to excessive ROS [16]. Besides, it has been demonstrated that APAP can induce endoplasmic reticulum (ER) stress and unfolded protein responses (UPR), which may lead to hepatotoxicity [17, 18]. Furthermore, other studies found that autophagy induced by APAP in the mouse liver and primary cultured hepatocytes accelerates APAP-induced liver cell death [19].

## 3. Animal Model of DILI Established by Antibacterial Agents

### 3.1. Antituberculosis Drug-Induced Liver Injury Models

Among antituberculosis medications, isoniazid (INH) is the most common one leading to DILI. In most cases, patients develop mild liver injury, while others have a severe phenotype that can progress to liver failure due to the production of anticytochrome P450 (CYP) antibodies [20]. One study has shown that high doses of INH (200 and 400 mg/kg/day) by gavages for one week produced steatosis in rats and increased sorbitol dehydrogenase (SDH), which indicated mitochondrial injury. However, lower dose for longer time of exposure did not induce liver steatosis [21]. The toxicity of INH is mediated through its metabolite, hydrazine, which is formed when it proceeds along the amidase pathway [22]. Hydrazine is considered to induce steatosis by altering liver gene expression profiles that promote production and transport of hepatic lipid [23]. Previous studies suggested that oxidative stress was also a possible mechanism responsible for toxicity and that CYP2E1 might be the important factor of oxidative stress and ROS production, leading to hepatotoxic injury [24]. It has been reported that acute liver injury caused by INH may be closely related to free radical lipid peroxidation. [24] Even though rats are commonly used for DILI model, one study suggested that it was unlikely to show the same hepatotoxicity as in human [25] because more covalent binding and higher serum concentration of INH in the liver of mice were observed than in rats, and the former process is more similar to that occurs in humans [21]. Therefore, mouse may be a better choice to generate an animal model of INH-induced liver injury.

Rifampicin (RIF), which is commonly used in combination with INH for tuberculosis, is not hepatotoxic itself, but it may occasionally cause dose-dependent interference with bilirubin uptake due to competition with bilirubin for clearance at the sinusoidal membrane, resulting in mild, asymptomatic unconjugated hyperbilirubinemia or jaundice without hepatocellular damage [22]. Besides, when used in combination with INH, RIF can accelerate metabolism of INH, produce toxic metabolites, and thus lead to worse liver damage [26]. Coadministration of INH (75 mg/kg) and RIF (150 mg/kg) by gavages once daily for one week resulted in obvious liver injuries including fatty accumulation, hepatic apoptosis, and the elevation of serum alanine aminotransferase (ALT) [27]. Wistar albino rats were used as animal models that were orally administered with 100* *mg/kg of body weight of INH and RIF [28]. Another study produced DILI models in rabbits by using INH (50 mg/Kg/d) alone or INH with RIF (100 mg/Kg/d) daily orally for 7 days. Rabbits receiving INH and RIF showed significant increase in serum ALT and AST levels, while those used INH alone showed no change [29]. Coadministration of INH and RIF can induce CYP 450 enzymes, significantly downregulating the expression of sodium taurocholate cotransporting polypeptide and bile salt export pump in liver [30], which indicates that RIF can promote hepatotoxicity caused by INH.

### 3.2. Tetracycline-Induced Liver Injury Models

Tetracycline is a broad-spectrum antibiotic closely associated with drug-induced hepatitis [31]. It is also well known to induce microvesicular steatosis and has a high risk to develop steatohepatitis, which is a rare form of liver injury leading to poor prognosis [32, 33].

The model was established with a single intraperitoneal injection of tetracycline at 50mg/kg [34]. Six hours later, histopathological analysis showed obvious microvesicular steatosis and a 2-fold increase of hepatic and serum triglyceride (TG) levels. Tetracycline affects cellular lipid metabolism by the following 4 major steps: (1) increased fatty acid uptake by upregulation of CD36 [34], (2) inhibition of fatty acid *β*-oxidation [35], (3) upregulation of genes involved in TG and cholesterol synthesis [36], and (4) inhibition of microsomal triglyceride transfer protein (MTP) activity to decrease the formation of TG-rich very-low-density lipoproteins particles in the microsomal lumen and inhibit hepatic lipoprotein secretion [32].

In another modeling method, male SD rats were injected intraperitoneally with 200 mg/kg tetracycline in saline [37]. Levels of intrahepatic triacylglycerol (IHTG), intrahepatic total cholesterol (IHTC), hepatic malondialdehyde (MDA), and serum ALT and AST increased significantly in those rats. A mice model receiving a similar treatment showed additionally extensive apoptosis in liver tissue. Researchers also found that the levels of the ER stress gene (IRE-l, ATF6, CHOP, and GRP78) transcripts were increased, indicating that tetracycline injection in mice induced hepatic apoptosis and ER stress [38]. A general view is that the increased influx of fatty acid into the livers is the first hit and oxidative stress due to lipid overload and attack of functional proteins is the second hit of tetracycline-induced microvesicular steatosis [37]. Subsequently, the overload lipid in hepatic cytoplasm activates the long-chain-fatty-acid-CoA ligase 1 and promotes the production of triacylglycerol or transportation of fatty acids into mitochondria, which accelerate *β*-oxidation and induce the stress of mitochondrial respiration chain and the high levels of ROS [37]. Researchers found that 26 targeted proteins might contribute to oxidative stress, most of which are located in mitochondria. The long-chain specific acyl-CoA dehydrogenase (ACADL) is specially identified in the tetracycline group that catalyzes the initial step in the chain shortening oxidation of fatty acids in mitochondria [39]. The reduction of ACADL activation may be responsible for the microvesicular fatty liver [37].

## 4. Animal Model of DILI Established by Antirejection Immunosuppressant Drug

Cyclosporine A (CsA), an immunosuppressant, is often used in the treatment of immune rejection and autoimmune diseases after organ transplantation. Studies have shown that CsA has dose-dependent hepatotoxicity and is related to its serum concentration [40, 41]. One study showed that administration of CsA increased levels of AST, ALT, and bilirubin, which represents functional liver damage [42].

In Hagar's experiment, Wistar rats CsA hepatotoxicity was induced by subcutaneous injection of CsA at a dose of 20 mg/kg body weight (Sandimmun infusion dissolved in olive oil to a final concentration of 25 mg/ml) daily for 21 days [43]. It has been acknowledged that CsA generates reactive oxygen species and lipid peroxidation [44]. A decline in GSH, GSH-Px, and catalase concentrations suggested a role of oxidative stress in CsA hepatotoxicity. Accumulation of ROS activates the defensive mechanism of hepatic cells through a variety of antioxidant enzymes, among which GSH, GSH-Px, and catalase have the most obvious impact [45]. Moreover, the depletion of GSH can promote CsA-induced hepatotoxicity [46]. Besides, increased concentration of thiobarbituric acid reactive substances (TBARS), which was associated with the initiation of CsA hepatotoxicity* in vitro* [43], indicated free radical attack on lipids due to lipid peroxidation. Therefore, the major reason of CsA hepatotoxicity is the imbalance between oxygen free radical generation and antioxidant system* in vivo*.

## 5. Animal Model of DILI Established by Traditional Chinese Medicine Drugs

### 5.1. Tripterygium wilfordii-Induced Liver Injury Models

*Tripterygium wilfordii* Hook F (TwHF) is a traditional Chinese medicine with anti-inflammatory, analgesic, and immune suppressive effect [47]. Triptolide (TP) is an important bioactive ingredient of TwHF and has a variety of pharmacological activities [48], for instance, immune modulation, antiproliferation, and anti-inflammatory. However, its severe hepatotoxicity limits the clinical application [49]. Cells apoptosis and mitochondria damage are the main mechanism of TP-induced liver injury. The mRNA expression of Nrf1, a main factor which is involved in mitochondrial regulation of cell apoptosis, was inhibited by TP. The downstream genes such as the mitochondrial transcription factor A (TFAM) and cytochrome C (Cyt-C) were also suppressed by TP [50]. What is more, Nrf2, as a nuclear transcription factor, was translocated into the nucleus to activate the target protective gene transcription [51, 52]; so the protein levels of cytoplasmic Nrf2 decreased, while the nuclear Nrf2 expression was induced after the TP treatment, which means that Nrf2 activators can be developed for therapeutic use [52]. Besides, the TP-induced liver damage could be observed in human cells* in vitro*. TP-induced apoptosis in L-02 cells, a normal human liver cell line, is related to increased expression of p53 and Bax protein, decreased Bcl-2 protein, loss of mitochondrial membrane potential, releases of Cyt-C from the mitochondrial intermembrane space toward the cytosol, and proteolytic activation of caspase 9 and caspase 3 [53]. Additionally, it is revealed that the mechanism of TwHF -induced liver injury is related to lipid peroxidation reaction.

TP-induced liver injury is dose-dependent [54, 55], time-dependent [56], and sex-related [54, 57, 58]. One researcher reported that 18 hours after mice were administered with 300mg/kg (20-fold of the common dose) by gavages, significant liver injury was observed [59]. The advantage of this modeling method is low death rate after modeling. When given orally at 400 *μ*g/kg/day for 28 days, Sprague-Dawley (SD) rats showed sex-related liver toxicity. Due to CYP3A2, a male-predominant enzyme considered to be responsible for sexual dimorphic metabolism of TP, male rats showed much less extent of liver injury compared to female ones [57]. Since sex is a fundamental factor that should not be ignored, female rats are more suitable to establish a model.

In a recent study [55], liver injury was induced in the female C57BL/6 mice through intragastric gavages with TP at a dose of 600 *μ*g/kg per mouse for 1, 3, or 5 days. Continuous TP administration led to significantly increased ALT and AST levels, which began to increase since day 1 and lasted until day 5. This finding suggested that TP-induced hepatotoxicity was dose-dependent, and hepatic natural killer (NKT) cells play a critical role in the development and progression of TP-induced liver injury. TP can activate NKT cells, dominantly releasing Th1 cytokine IFN-*γ*, recruiting macrophages and neutrophils, and resulting in liver injury.

*Tripterygium wilfordii* multiglycoside (GTW), another extract derived from TwHF, was applied to treat the rheumatoid arthritis and other immune diseases in China. One study suggested that a high dose of 120 mg/kg/day for 20 days given to female Wistar rats by gavages could lead to liver injury [54]. The rats showed a significant reduction of food intake and body weight, elevation of the relative liver weight, classic histopathological changes, and reduction of the serum ALB and total protein levels. Histopathology showed that there is partial necrosis with inflammatory cell infiltration in hepatocytes. The researchers deduced that GTW might cause oxidative stress in the liver cells, leading to cell dysfunction and even apoptosis or necrosis. Expression of hepatic genes involved in certain cellular pathways was also downregulated particularly with regard to metabolic pathways, the peroxisome proliferator-activated receptor (PPAR) signaling pathway, and cellular stress. Interestingly, the study noted that the classical indicator of liver function, the ALT level, was not suitable for evaluating liver injury in the experiment because it distributes widely besides liver tissues, whereas the level of ALB can more sensitively reflect biosynthesis in the liver and liver damage than ALT, since it is predominantly synthesized by the liver and has a short half-life.

### 5.2. Polygonum Multiflorum-Induced Liver Injury Models

Polygonum multiflorum (PM), known as Heshouwu in China, is a traditional Chinese medicinal herb for many diseases [60]. However, the major hepatotoxins in PM remain controversial. Some studies indicated that highly reactive anthraquinones formed in the colon lead to hepatotoxicity [61, 62], while others believed that it was correlated with the content of tetrahydroxystilbene glucosides [63]. Ethanol-eluted extract, including emodin, inhibits the growth of hepatic L-02 cells [64] and it is possible to suppress cell proliferation and promote cell apoptosis by inhibiting the activation of signaling molecules such as signal transducers and activators of transcription (STAT) [65] and reducing UGT1A8 mRNA expression products when interacting with stilbene glucoside [66].

One report has demonstrated that administration of isolated extract of PM, the chloroform extract (CH), the ethyl acetate extract (EA), and residue (RE) to normal rats failed to induce significant liver injury [67]. Treatment with lipopolysaccharide (LPS) alone only caused slight inflammatory reaction. However, when EA is combined with a small dose of LPS, it can specifically result in hepatotoxicity, which means that LPS and EA extract can be used as inducers to establish PM-induced liver injury model. Followed by tail vein injection of LPS (2.8 mg/kg) 3 hours later, the male SD rats were intragastrically administered EA of PM. The investigation of PM-induced liver injury was comprehensive, and the doses of the extracts for modeling were not fully determined. The ALT and AST activities in blood and in portal areas were remarkably increased 7 hours after modeling. Hepatocyte focal necrosis, loss of central vein intima, and a large number of inflammatory cells' infiltration were also observed. The inflammation caused by LPS increases the susceptibility to the toxicity from other chemicals [68]. It was reported that EA affected the activity of mitochondrial enzymes related to TCA cycle in liver, possibly disturbing the metabolism. Also, the decreased serum level of creatine in the EA/LPS group suggested steatosis, which could exist in the nonalcoholic fatty liver disease samples of human and other animals [69, 70].

Many other researchers have investigated different extracts of PM. In Lin's experiment [71], the rats were treated orally with different extracts of PM at doses of 6 g/kg per day, lasting for 90 days. The results exhibited that all the extracts including water, 30% ethanol, 70% ethanol, dichloromethane (DCM), and total extracts induced hepatotoxicity, especially the 30% ethanol group and DCM group. However, the hepatotoxic mechanisms of various extracts were different. The oxidative phosphorylation pathway is the possible mechanism and NADH dehydrogenase family proteins and Slc16a2 may be potential biomarkers of hepatotoxicity due to PM. In Wu's experiment [63], the Kunming mice were fed with water decoction and acetone extracts of raw and processed PM at the doses of 5,10, and 20 g/kg per day for 28 days, which were equivalent to 10, 20, and 40 times of the upper dosage for human recommended in Chinese Pharmacopoeia (0.5 g/kg).The result indicated that the toxicity of PM decreases significantly after being processed, increased in proportion to the dosages, and does not depend on the content of anthranoid derivatives and that the toxicity of the aqueous decoction was much higher than that of the acetone extract. Another similar experiment [72] established rat models at the doses of 30 g/kg and demonstrated that PM induces the metabolic disorders of energy metabolism, amino acid and lipid metabolism, which indicated liver injury. Huang's experiment [73] also showed that the liver damage was more severe as the dose increased. The SD rats were continuously treated with 5.40 g/kg/d water extract of processed PM by intraperitoneal administration for 7 days. The results showed that AST was decreased, while ALT was increased. Meanwhile, it was also found that CYP1A2 and CYP2E1 mRNA expression levels were significantly inhibited in the liver of rats.

The above models were established using different extracts from traditional Chinese medicine. The composition of traditional Chinese medicine is complex, and thus it is recommended to adopt appropriate methods based on different ingredients and research aims.

## 6. Animal Model of DILI Established by Antiepileptic Drugs

### 6.1. Sodium Valproate-Induced Liver Injury Models

Sodium valproate (valproic acid, VPA) is a commonly used antiepileptic drug that can lead to severe liver injury and even liver failure. Overdose of VPA may cause acute hepatocellular injury, even in the absence of preexisting liver disease [74].

Adult male SD rats were given 500mg/kg/d of VPA by gavages for 2 weeks to establish liver injury model [75, 76]. VPA-treated rats showed significant increase in the activities of AST, ALT, and alkaline phosphatase. The mechanism is still unclear [77], but it may be related to the interference with the mitochondrial beta oxidation of fatty acids. Previous studies have reported that treatment of rat hepatocytes with VPA leads to increased oxidative stress, as measured by elevated levels of 15-F2t-IsoP [78]. Decreased GSH levels also demonstrated that VPA-induced tissue injury is associated with increased oxidative stress. This model can be used both in investigation of liver injury mechanisms and in evaluation of medicine effect.

### 6.2. Carbamazepine (CBZ)-Induced Liver Injury Models

Among the classic four major classes of commonly used antiepileptic drugs [phenobarbital, phenytoin sodium, VPA, and carbamazepine (CBZ)], liver damage caused by CBZ is less common than VPA. Liver biopsies of patients receiving CBZ therapy were compatible with hepatotoxic damage, and the symptoms were reversible with medication withdrawal [79]. The hepatotoxicity generally appears as two forms: (1) a granulomatous hepatitis with fever and liver dysfunction and (2) an acute hepatitis and hepatocellular necrosis with inflammation [80, 81]. What is worth mentioning is that some other drugs, for instance, VPA, phenytoin, lamotrigine, and felbamate, can elevate the concentrations of some active derivatives of CBZ and further increase their hepatotoxicity [82, 83].

One study by Higuchi et al. [84] showed that a single administration of CBZ could not induce liver injury at any of the experimental doses, and repeated administration of CBZ is necessary to establish CBZ-induced liver injury model. Male BALB/c mice were orally administered CBZ at a dose of 400 mg/kg for 4 days and 800 mg/kg on the 5th day to generate DILI model. As a result, the plasma AST and ALT levels were significantly increased 24 and 48 hours after the last CBZ administration with prominent hepatic necrosis and loss of hepatocytes, especially around the central vein. The underlying mechanisms behind CBZ-induced liver injury may be related to the arene oxide metabolite for the hapten formation and a subsequent involvement of the immune system [85]. Eghba's study showed that CBZ can induce oxidative stress, forming increasing ROS and lipid peroxidation products, while decreasing mitochondrial membrane potential [86]. CBZ is metabolized in hepatocytes by CYPs, and the reactive metabolite(s) induce ROS production in macrophages, where danger signals are released to activate toll-like receptor 4 (TLR4) and the receptor for advanced glycation end product (RAGE) [87]. The activated TLR4 and RAGE lead to the secretion of chemokines and proinflammatory cytokines, which result in inflammation in the liver. The necrotic hepatocytes secrete the ligands of TLR4 and RAGE, inducing further inflammation and chemokines in the liver [84, 88]. IL-17 induced by CBZ, which was reported to participate in various immune mediated hepatotoxicity in mice [84, 89], also reduced the plasma AST and ALT levels and MPO-positive cells in the liver.

## 7. Animal Model of DILI Established by Antithyroid Drugs 

Methimazole (MMI) and propylthiouracil (PTU) have been used in the management of hyperthyroidism for more than half a century. However, hepatotoxicity is one of the most deleterious side effects associated with these medications [90]. Currently, it is not common to establish liver injury model using antithyroid hormone, but the liver injury caused by this drug cannot be overlooked, especially in children [91]. The mechanisms of hepatic injury induced by antithyroid drugs may be a combination of drug reactive metabolite formation and immunological reactions [92], and Kupffer cell-mediated immune responses are crucial factors for the exacerbation of MMI-induced liver injury in rats [93].

One report indicated a synergistic liver injury from antithyroid drugs and LPS coexposure. [94] Mice were treated with a nonhepatotoxic dose of LPS (100 *µ*g/kg, i.p.) or its vehicle. Nonhepatotoxic doses of MMI (10, 25, and 50 mg/kg, oral) and PTU (10, 25, and 50 mg/kg, oral) were administered two hours after LPS treatment. The results showed that liver injury was evident only in the LPS and MMI/PTU groups, resulting in alteration to hepatotoxicity biomarkers and histopathological changes in liver tissues, which was consistent with former studies [95, 96]. Liver may become more sensitive to injury due to inflammatory stress, and LPS can stimulate the inflammation by activating TLR and Kupffer cells [97], which may produce harmful mediators and attract other inflammatory cells to the liver tissues [98]. Moreover, it has been reported that myeloperoxidase (MPO)-mediated MMI metabolism could lead to oxidative stress and glutathione depletion* in vitro*. [99]

Currently, there are few studies on the hepatotoxicity of antithyroid drugs, and the modeling methods need to be further explored.

## 8. DILI Models of Drug/Inflammation Interaction

Idiosyncratic drug-induced liver injury (iDILI) is a rare disorder that is not associated directly with dose or duration of the drug and little is known about the mechanism [100]. There are compelling evidences supporting the inflammatory stress hypothesis that inflammation increases the susceptibility of tissues to toxic substances, causing individuals to develop toxic reactions at nontoxic doses [101, 102].

Researchers have built several iDILI animal models based on drug/inflammation interaction theory. Mice were treated with trovafloxacin (150 mg/kg; p.o.) and then 3 hours later with LPS (67×10^6^ EU/ kg; i.p.), which resulted in elevation of plasma ALT activity by 9 h after trovafloxacin /LPS coexposure and peak of plasma ALT at 15 to 21 hours after LPS [103]. In a sulindac/LPS model, rats were given two administrations of sulindac (50 mg/kg, p.o.) with a 16-hour interval, and half an hour before the second administration of sulindac, a nonhepatotoxic dose of LPS was administered (8.25 × 10^5^ EU/kg, i.v.) to rats. Liver injury was most obvious 12 hours after sulindac /LPS coexposure in rats [104]. Rats were pretreated with LPS (29×10^6^ EU/kg. i.v.) and given Diclofenac (DCLF, a NSAID, 20 mg/kg, i.p.) 2 hours later, causing a significant increase in serum ALT activity compared with the control group. However, LPS alone or low doses of DCLF (less than 40 mg/kg) did not cause changes in serum ALT activity [95]. It is a compelling finding because most patients take NSAIDs to treat inflammation-related diseases, and thus the interaction between drug and inflammation may worsen liver damage. Higher levels of genes related to inflammation expressed in the LPS/DCLF group and the GO functional analysis indicated that polymorphonuclears (PMNs) might participate in the pathogenesis. Ranitidine (RAN) was also used to establish the iDILI model [105]. Rats fasted for 24 hours were given LPS (44.4×10^6^ EU/kg, i.v.) 2 hours before RAN (30 mg/kg, i.v.). Cotreatment of LPS/RAN resulted in a 6- to 10-fold increase in ALT and a 7- to 14-fold increase in AST activity, while GGT increased by 1.5-fold. Acute moderate hepatic necrosis, hepatic cytoplasmic eosinophilia, and nuclear pyknosis occurred in the cotreated group, and invasive PMN was present in the necrotic foci. These data indicated that iDILI is more likely to be liver cell damage than cholestatic injury. The above effect was not significant when RAN or LPS was administered alone.

LPS activates TLR4 on Kupffer cells, and cytokines such as TNF-*α*, IL-1, and IL-6 are upregulated, leading to the imbalance of pro-/anti-inflammatory response and damage to liver tissues [97]. Additionally, TNF-*α* and IL-1 increase plasminogen activator inhibitor-1 (PAI-1), which inhibits the generation of plasmin and reduces fibrinolytic activity of endothelial cells [106, 107]. It was observed that the plasma levels of thrombin-antithrombin III complex (TAT) and PAI-1 were elevated in rats given LPS [104], proving that inflammatory response can activate the coagulation system, which is critical in the pathogenesis of liver [108]. Furthermore, PMNs occur in inflammatory infiltrates and are involved in hepatotoxic response to LPS probably through releasing cytotoxic factors [109, 110]. Hepatocytes are more sensitive to these cytotoxic factors after receiving certain drugs.

## 9. Conclusion

In this article, we have reviewed the current established animal models of DILI, especially the approaches, characteristics, and possible mechanisms of rodent models (the above information were summarized in Table 1). Besides, we elaborated the mechanisms and useful animal models related to drug/inflammation interaction, which is an interesting theory in DILI. Despite the extensive research achievements, DILI still remains a clinical challenge due to the poorly predictable outcomes. Animal models contribute to researches on mechanisms and protective drugs. Various drugs are able to generate animal models of DILI, and different methods of administration of the same drugs may lead to different outcomes, such as through gavages and intraperitoneal injection. Some of the methods have been fully developed and widely used in DILI research, for instance, acetaminophen-induced hepatotoxicity. Others, including gradients of traditional Chinese medicine and some antithyroid drugs, are less commonly used and the mechanisms need further digging. Meanwhile, attention should also be paid to drug incompatibility, which may aggravate the hepatotoxicity. In addition, sex of the animal may also influence the hepatotoxicity. Therefore, researchers can choose different methods based on the study purpose and the features of different models.

Emerging approaches to investigate the underlying mechanisms of DILI have not been fully introduced and applied in animal models, for instance, genomics, proteomics, and metabolomics related studies, which might be the future direction in this research field. Finally, even though animal studies can predict about 70% of human hepatotoxicity, the animal researches cannot provide a full prediction of human outcomes. Before the study results are applied in human body, more validation researches are required to confirm the effect observed in animal models. 

## Figures and Tables

**Table 1:** Summary of modeling methods, pathological features, and molecular mechanisms of DILI.

Drug	Modeling methods	Pathology features	Molecular mechanisms
APAP [10, 11]	*Dose*: 300-500 mg/kg *Administration*: Single i.p., observe 4 hours later	Sinusoidal congestion and hemorrhage, dilated central vein, inflammatory cell infiltration, degenerated hepatocytes showing perinuclear vacuolization	1. GSH depleted by NAPQI 2. Mitochondrial dysfunction 3. Oxidative stress 4. Activating the protein kinase JNK 5. Hepatocyte apoptosis 6. ER stress and UPR

INH [21, 27]	1. *Dose*: 200 or 400 mg/kg/day *Administration*: Gavage daily for one week 2.* Dose*: INH 75 mg/kg/day and RIF 150 mg/kg/day *Administration*: Gavage daily for one week	Hepatocyte steatosis and edema, the sinus almost disappears, part of the mitochondrial cristae disappeared, and the endoplasmic reticulum was vesicular	1. Mitochondrial injury and dysfunction 2. Hydrazine, the toxic metabolite 3. Apoptosis 4. Oxidative stress 5. Coadministration of RIF induces CYP 450 enzymes and promotes hepatotoxicity 6. Free radical lipid peroxidation

Tetracycline [34, 37]	1. *Dose*: 50 mg/kg *Administration*: Single i.p., observe 6 hours later 2. *Dose*: 200 mg/kg in saline *Administration*: Single i.p., observe after 36-hour free diets and 12-hour diet deprivations	Hepatic parenchymal cells microvesicular steatosis, hydropic degeneration around the pericentral zone	1. Affecting cellular lipid metabolism 2. Apoptosis 3. ER stress 4. Oxidative stress

CsA [43],	*Dose*: 20 mg/kg, (Sandimmun infusion dissolved in olive oil, 25mg/ml) *Administration*: Subcutaneous injection daily for 21 days	Hepatocyte steatosis, apoptosis, vacuolar degeneration hepatocytes, lipid droplets, reduced mitochondrial cristae, and rough endoplasmic reticulum cystic expansion	1. Imbalance between production of oxygen free radicals and the endogenous antioxidant defense system 2. Substantial increase in caspase 3 activity that induces apoptosis

TwHF [54, 55, 59]	1. *Dose*: 300 mg/kg TP *Administration*: Gavage, observe after 18 hours 2. *Dose*: 600 *μ*g/kg TP *Administration*: Intragastric gavage daily for 5 days 3. *Dose*: 120 mg/kg GTW *Administration*: Gavage daily for 28 days	Extensive hepatocyte turbidity, focal hepatocyte ballooning in the central vein and peripheral areas, scattered eosinophilic changes in hepatocytes Tendencies toward augmented focal necrosis, inflammatory cell infiltration, and bile duct hyperplasia Partial necrosis with inflammatory cell infiltration in hepatocytes	1. Cells apoptosis 2. Mitochondria lesions 3. Immune response 4. Lipid peroxidation 5. Inflammation 6. Oxidative stress

PM [67, 73]	1. *Dose*: 2.8 mg/kg LPS with uncertain doses of EA extract of PM *Administration*: Tail vein injection of LPS and intragastrically administer EA extract of PM 2. *Dose*: 5.4 g/kg water extract of processed PM *Administration*: i.p. for 7 days	Hepatocyte focal necrosis, loss of central vein intima and a large number of inflammatory cell infiltration	1. Disruption of energy metabolism, amino acid and lipid metabolism 2. Inflammatory response 3. Steatosis 4. CYP1A2 and CYP2E1 mRNA expression levels were significantly inhibited

VPA [75, 76]	*Dose*: 500 mg/kg/d *Administration*: Gavage for 2 weeks	Massive necrosis, liver steatosis and increase of lipid accumulation	1. Oxidative stress 2. Hepatotoxic metabolites

CBZ [84]	*Dose*: 400 mg/kg for 4 days and 800 mg/kg on the 5th day *Administration*: Oral gavage	Prominent hepatic necrosis and loss of hepatocytes, especially around the central vein Hepatocytes showed hemorrhage, centrilobular and sinusoidal congestion	1. The neutralization of IL-17 2. Metabolite(s) indirectly activates TLR4 and RAGE, resulting in inflammation

Drug/inflammation interaction models	*MMI or PTU with LPS* [94] *Dose*: MMI, 10-50 mg/kg, PTU, 10-50 mg/kg, and LPS, 100 *µ*g/kg *Administration*: MMI&PTU: oral, and LPS: i.p.	Inflammatory cells infiltration, intracanalicular cholestasis, fatty changes	1. Drug reactive metabolite formation and inflammation induction 2. Immunological reactions 3. Oxidative stress
	*TVX with LPS* [103] *Dose: *TVX: 150 mg/kg, LPS: 67×10^6^ EU/ kg *Administration*: TVX: oral, and LPS: i.p.	Inflammatory cell infiltration; coagulative necrosis located predominantly midzonally and in centrilobular region	1. Enhanced TNF release 2. Activation of the hemostatic system 3. Neutrophils accumulation
	*SLD with LPS* [104] *Dose: *SLD: 50 mg/kg, LPS: 8.25 × 10^5^ EU/kg *Administration: *SLD: oral; two administrations with a 16-hour interval. LPS: i.v.; half an hour before the second administration of SLD	Midzonal hepatic necrosis	
	*DCLF with LPS* [95] *Dose: *DCLF: 20 mg/kg, LPS: 29 × 10^6^ EU/kg *Administration: *DCLF: i.p., and LPS: i.v., 2 hours before DCLF	Parenchymal edema (multifocal); parenchymal hemorrhage (multifocal); apoptosis (random); leukocyte infiltration	
	*RAN with LPS* [105] *Dose: *RAN: 30mg/kg, LPS: 44.4 × 10^6^ EU/kg *Administration: *RAN: i.v., and LPS: i.v., 2 hours before RAN	Acute, multifocal, midzonal hepatic necrosis developed as early as 3 h; hepatocellular cytoplasmic eosinophilia and nuclear pyknosis; variable numbers of infiltrating PMNs	

i.p., intraperitoneal injection; i.v., intravenous injection; APAP, Acetaminophen; INH, Isoniazid; CsA, Cyclosporine A; TwHF, *Tripterygium wilfordii*; TP, Triptolide; GTW, *Tripterygiumwilfordii* multiglycoside; PM, Polygonum multiflorum; VPA, Sodium valproate (valproic acid); CBZ, carbamazepine; NAC, *N*-acetyl cysteine; MMI, Methimazole; PTU, propylthiouracil; LPS, Lipopolysaccharide; TVX, Trovafloxacin; SLD, Sulindac; DCLF, Diclofenac; RAN, Ranitidine.
